# Species-Specific and Pollution-Induced Changes in Gene Expression and Metabolome of Closely Related *Noccaea* Species Under Natural Conditions

**DOI:** 10.3390/plants13223149

**Published:** 2024-11-09

**Authors:** Valentina Bočaj, Paula Pongrac, Sina Fischer, Matevž Likar

**Affiliations:** 1 Biotechnical Faculty, University of Ljubljana, Jamnikarjeva 101, SI-1000 Ljubljana, Slovenia; valentina.bocaj@bf.uni-lj.si (V.B.); paula.pongrac@bf.uni-lj.si (P.P.); 2 Jožef Stefan Institute, Jamova 39, SI-1000 Ljubljana, Slovenia; 3 School of Biosciences, University of Nottingham, Loughborough LE12 5RD, UK; sina.fischer@nottingham.ac.uk

**Keywords:** *Noccaea praecox*, *Noccaea caerulescens*, glutathione, phenylpropanoid pathway, flavonoid biosynthesis

## Abstract

Hyperaccumulators within the *Noccaea* genus possess many promising genetic and metabolic adaptations that could be potentially exploited to support phytoremediation efforts and/or crop improvement and biofortification. Although hyperaccumulation is very common in this genus, individual species display specific traits as they can accumulate different elements (e.g., zinc, cadmium, and/or nickel). Moreover, there appears to be some populational variability with natural selection increasing the metal tolerance in metallicolous populations. Therefore, employing robust methods, such as integrated analysis of the transcriptome and metabolome, is crucial for uncovering pivotal candidate genes and pathways orchestrating the response to metal stress in *Noccaea* hyperaccumulators. Our study highlights several species-specific traits linked to the detoxification of metals and metal-induced oxidative stress in hyperaccumulating *N. praecox* when compared to a closely related model species, *N. caerulescens*, when grown in the field. Transcriptome analysis revealed distinct differences between the three studied natural *Noccaea* populations. Notably, we observed several pathways frequently connected to metal stress, i.e., glutathione metabolism, phenylpropanoid biosynthesis, and flavonoid biosynthesis, which were enriched. These differences were observed despite the relative evolutionary closeness of studied species, which emphasizes the importance of further expanding our knowledge on hyperaccumulators if we want to exploit their mechanisms for phytoremediation efforts or food quality improvements.

## 1. Introduction

Over the recent decades, environmental contamination with heavy metals, including cadmium (Cd), lead (Pb), copper (Cu), and zinc (Zn), has increased at an alarming rate. This contamination not only hampers growth and development in both plant and animal life but also poses a significant threat to human health, emerging as a critical environmental challenge [1,2,3,4]. However, these polluted habitats and naturally occurring polluted soils host an extraordinary group of plants that can cope with toxic concentrations of heavy metals. Among these are hyperaccumulators, plants capable of accumulating unusually high concentrations of toxic metals in their tissues [5], offering a promising avenue for mitigating the issue of metal pollution. Furthermore, they possess many promising genetic and metabolic adaptations that could be potentially exploited for crop improvement and biofortification [6].

The genus *Noccaea* includes several hyperaccumulator species, e.g., *N. goesingense*, *N. caerulescens*, and *N. praecox*, capable of accumulating high concentrations of metals in their shoots. Studies have shown that the Ni hyperaccumulation in *N. goesingense* is due to its tolerance of the metal rather than a difference in translocation rates compared to non-hyperaccumulators [7]. Similarly, *N. caerulescens*, a Cd and Zn hyperaccumulator, has been found to possess genes that confer heavy metal tolerance and play a role in metal hyperaccumulation [8]. The significance of antioxidative defenses, particularly catalase activity, in the Cd tolerance of *N. caerulescens*, has also been highlighted [9]. In addition, analysis of the spatial distribution of metals in *N. praecox* leaves revealed preferential localization of toxic metals away from important metabolic processes, e.g., vacuoles and apoplast [10]. These findings collectively indicate that hyperaccumulation is a complex phenomenon that requires an interplay of metal tolerance mechanisms and processes that accelerate metal uptake.

Although hyperaccumulation is a moderately common trait in the genus *Noccaea*, there seems to be some populational variability as natural selection was observed to have increased metal tolerance in metallicolous populations of *N. caerulescens* [11,12]. A series of studies have explored the transcriptome of plant hyperaccumulators under metal stress. Zhao et al. (2021) [13] found that the hyperaccumulator *Phytolacca americana* up-regulates genes related to lignin biosynthesis, heavy metal accumulation, sulphur metabolism, and glutathione metabolism in response to Cd stress. This suggests the plant may increase the lignification of its cell wall and increase thiol compounds to detoxify Cd. Similarly, transcriptome analysis was used to explore the molecular mechanisms of metal hyperaccumulation and hypertolerance in *N. caerulescens* [14] and *N. goesingense* [15]. They identified significant variations in gene expression profiles among different accessions, providing insight into the underlying molecular mechanisms. These studies highlight the importance of comprehensively understanding the effects of metal hyperaccumulation on the plant transcriptome and metabolome. In addition, it is important to consider comparisons between different hyperaccumulators as they could help us reject or support hypotheses based on single-species observations and resolve the basis of whether any specific tolerance or accumulation mechanism is species specific or more universal. Leveraging this knowledge could lead to the development of strategies for improving phytoremediation efforts, crop tolerance to metal stress, and biofortification attempts.

The widespread adoption of multi-omics analysis technologies has recently revolutionized the exploration of plant responses to abiotic stress. Transcriptomics and metabolomics are robust methodologies for thoroughly dissecting the biological processes within plants thriving in challenging environments [16,17]. In this study, we conducted an integrated analysis of the transcriptome and metabolome of *N. praecox*. Plants were studied in their natural environment under different metal conditions to uncover pivotal candidate genes and pathways orchestrating the response to metal stress. In addition, a naturally co-occurring population of *N. caerulescens* at the non-polluted site provided us with an opportunity to compare the results with a well-known and closely related hyperaccumulator growing under the same conditions. While indoor experiments offer controlled conditions to decipher the cause and consequences of gene expression, they do not provide real-life situations where plants interact with their entire phytobiomes. Our objective was, therefore, to uncover pivotal candidate genes and pathways orchestrating the response to metal stress in *N. praecox* grown in its natural environment, thereby paving the way for novel insights crucial for genetic engineering aimed at enhancing plant capabilities for phytoremediation applications or crop biofortification.

## 2. Results

**Figure 1:**
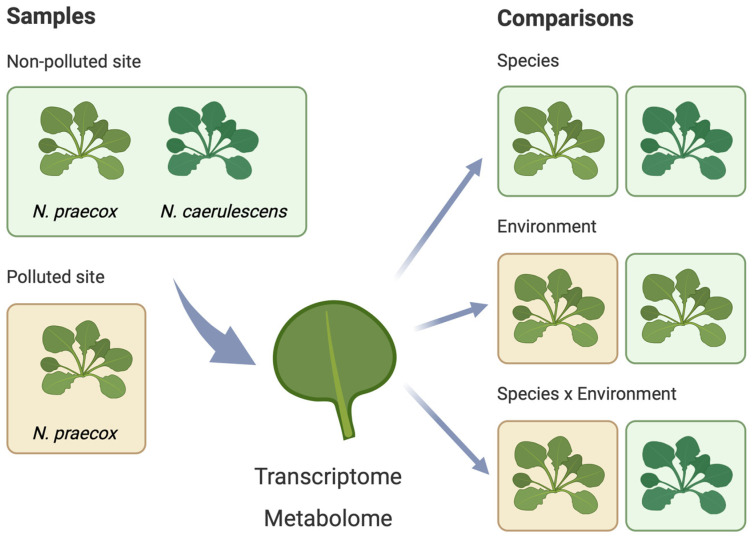
Schematic representation of the study.

Three different comparisons of gene expression and metabolites were made: (i) a between-species comparison at the non-polluted site, i.e., *N. praecox* and *N. caerulescens* (labelled genotype), (ii) a between-environment comparison of *N. praecox* at the polluted and non-polluted site (labelled environment), and (iii) a species × environment comparison of *N. praecox* at the polluted site vs. *N. caerulescens* at the non-polluted site (labelled species × environment) (Figure 1).

### 2.1. Metal Concentrations

Concentrations of Cd, Zn, and Pb were measured in the leaves of both *Noccaea* species, with some statistically significant differences (Table 1). The leaf concentrations of Cd and Pb were much higher in the leaves of *N. praecox* from the polluted site when compared to the *Noccaea* populations at the non-polluted site. In contrast, leaf Zn concentrations were comparable for *N. praecox* from the polluted site and *N. caerulescens* from the non-polluted site, whereas Zn concentration in leaves of *N. praecox* from the non-polluted site were much lower than in other populations.

### 2.2. Transcriptome Analysis Results

The initial Trinity assembly yielded 202,151 transcripts with an ExN50 of 1828 bp. The BUSCO score for the initial assembly against orthologs from Viridiplantae showed 98.1% complete, 1.4% fragmented, and 0.5% missing genes. The assembly showed a read mapped back to the transcriptome (RMBT) value of 67.7%.

**Figure 2:**
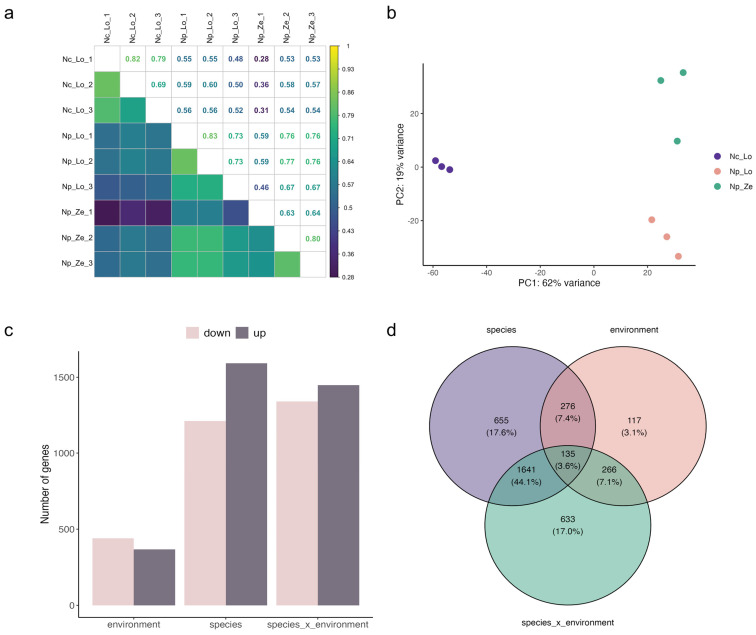
Transcriptome data of *Noccaea caerulescens* (Nc) and *N. praecox* (Np) from non-polluted (Lo) and polluted (Ze) sites. (**a**) Pearson correlation coefficient analysis, (**b**) PCA analysis, (**c**) number of differentially expressed genes (DEGs), and (**d**) Venn diagram of DEGs for species (Np_Lo vs. Nc_Lo), environment (Np_Ze vs. Np_Lo), and species_x_environment (Np_Ze vs. Nc_Lo) comparisons.

Correlation and PCA analysis demonstrated distinctive differences between the two studied species (*N. praecox* and *N. caerulescens*) collected at non-polluted and polluted sites (Figure 2a,b). The environmental comparison yielded 2811 DEGs, of which 1594 were up-regulated and 1217 were down-regulated, the species comparison of 2798 DEGs (1450 up-regulated and 1348 down-regulated, and the species × environment comparison of 809 DEGs (368 up-regulated and 441 down-regulated) (Figure 2c). Across all comparisons, 3723 DEGs were identified (Figure 2d), among which 135 (3.6%) were common to all three. Comparing the species and environmental effects yielded 633 and 655 unique DEGs, respectively, and 276 genes, which change in response to both factors. Comparing the genotype × environment and the other two contrasts yielded 117 unique DEGs (Figure 2d). Many more DEGs (1641) were common between the species and species × environment comparisons than the environment and species × environment comparisons (266) (Figure 2d).

**Figure 3:**
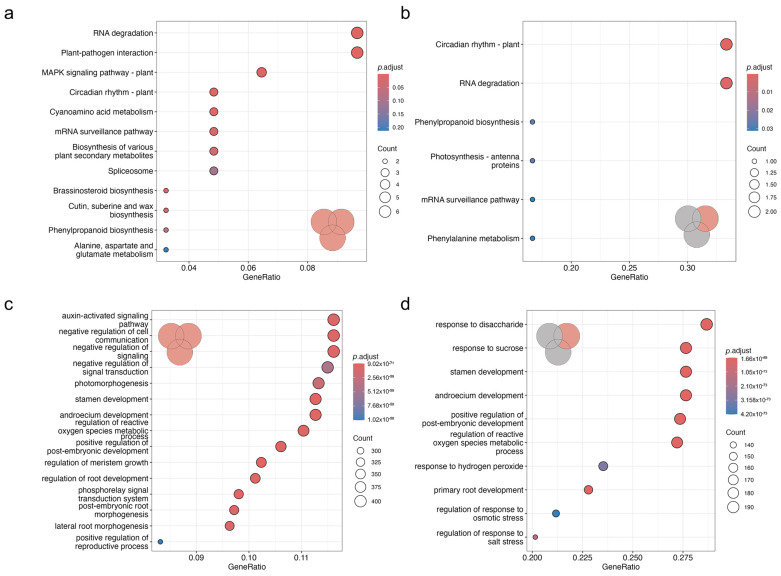
The enrichment analysis of KEGG functional pathways (**a**,**b**) and GO terms (**c**,**d**) among all DEGs (**a**,**c**) and DEGs in *N. praecox* grown in different environments (**b**,**d**). The inset Venn diagram depicts the differentially expressed genes used for pathway enrichment analysis, as depicted in Figure 2d. The colors of dots represent the adjusted *p* values and sizes represent the counts of transcripts in the category.

The GO terms and KEGG pathway enrichment analysis (Figure 3) showed the enrichment of several pathways. Among genes transcriptionally responsive to either environment or species or species × environment (Figure 3a,c), the significantly enriched KEGG pathways were RNA degradation, plant–pathogen interactions, and MAPK signaling pathway. The most enriched GO terms were gravitropism, negative regulation of cell, and negative regulation of signal transduction. In contrast, among genes exclusively responsive to a different environment (Figure 3b,d), significantly enriched KEGG pathways in *N. praecox* were those related to circadian rhythm, RNA degradation, phenylpropanoid biosynthesis, photosynthesis, mRNA surveillance pathways, and phenylalanine metabolism. In addition, several GO terms were enriched, including the regulation of reactive oxygen species and several other physiological, morphological, or developmental processes.

### 2.3. Metabolome Analysis Results

**Figure 4:**
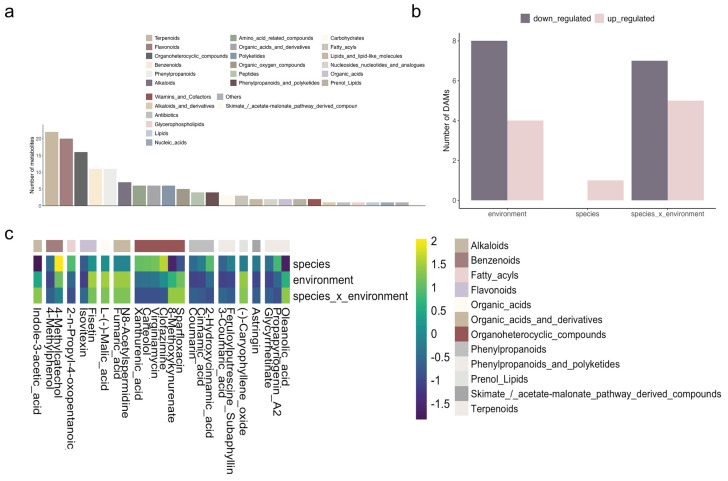
Metabolome analysis of *Noccaea praecox* (Np) and *N. caerulescens* (Nc): (**a**) the number of different metabolites; (**b**) the number of differentially accumulated metabolites (DAMs); (**c**) the heatmap of DAMs for species (Np_Lo vs. Nc_Lo), environment (Np_Ze vs. Np_Lo), and species_x_environment (Np_Ze vs. Nc_Lo) comparisons.

Metabolome analysis was performed to explore the metabolic response of *N. praecox* to metal stress and to compare it to *N. caerulescens*. All three groups were well distinguished by PCA analysis (Supplementary Figure S1). A total of 7252 metabolites were screened, with 1667 identified and 548 assigned to classes. Organoheterocyclic compounds, phenylpropanoids, and terpenoids comprised the groups with the highest number of metabolites identified (Figure 4a). Differential accumulation analysis revealed 26 differentially accumulated metabolites (DAMs) across all comparison groups. Specifically, in the environment and environment × species, there were 12 and 13 DAMs, respectively (Figure 4b). The number of DAMs was notably lower in species comparison, with no down-regulated DAMs and only one up-regulated DAM.

KEGG enrichment analysis identified phenylalanine metabolism, phenylpropanoid biosynthesis, and the citrate cycle as the most enriched pathways for between-environment comparison. Flavone and flavonoid biosynthesis, phenylalanine metabolism, and phenylpropanoid biosynthesis were the most enriched pathways in *N. caerulescens* compared to *N. praecox* plants from either location (Supplementary Figure S2).

When comparing the *N. praecox* population from a polluted site to the population from a non-polluted site (Figure 4c), the highest number of up-regulated DAMs was observed for flavonoids (fisetin), organic acids (malic acid), organic acids and derivatives (fumaric acid and N8-acetylspermidine), and prenol lipids ((−) caryophyllene oxide). The down-regulated DAMs were observed in benzenoids (4-methylphenol), flavonoids (isovitexin), phenylpropanoids (coumarin and cinnamic acid), shikimate pathway compounds (astringin), and terpenoids (glycyrrhetinate). In contrast, under non-polluted conditions, only 4-methylcatechol was up-regulated in *N. praecox* compared to *N. caerulescens*.

### 2.4. Results of Integrated View of Transcriptomics and Metabolomics

We found 70 DEGs and 11 DAMs up-regulated in 9 pathways when comparing *N. praecox* from the polluted and non-polluted sites (Supplementary Figure S3). Moreover, when comparing *N. praecox* from the polluted site with *N. caerulescens* from the non-polluted site, we observed 126 DEGs and 25 DAMs up-regulated across 16 KEGG pathways (Supplementary Figure S4). Similarly, comparing *N. praecox* and *N. caerulescens* growing at the non-polluted site revealed that 111 DEGs and 19 DAMs were up-regulated in 15 KEGG pathways (Supplementary Figure S5). In general, arginine and proline metabolism, carotenoid biosynthesis, phenylalanine metabolism, phenylpropanoid biosynthesis, and ubiquinone and other terpenoid–quinone biosynthesis pathways were up-regulated in the environment comparison. For the species comparison, the most up-regulated DEGs and DAMs are recorded for biosynthesis of secondary metabolites; alanine, aspartate, and glutamate metabolism; amino sugar and nucleotide sugar metabolism; glycolysis/gluconeogenesis; phenylpropanoid biosynthesis; glutathione metabolism; and purine metabolism. Notably, the amino sugar and nucleotide sugar metabolism, glycolysis/gluconeogenesis, and phenylpropanoid biosynthesis were characteristic of *N. praecox* vs. *N. caerulescens*, although the phenylpropanoid biosynthesis showed less up-regulated functions when comparing *N. praecox* between polluted and non-polluted conditions.

### 2.5. DEGs and DAMs Connected with Metal Response

#### Glutathione Metabolism

**Figure 5:**
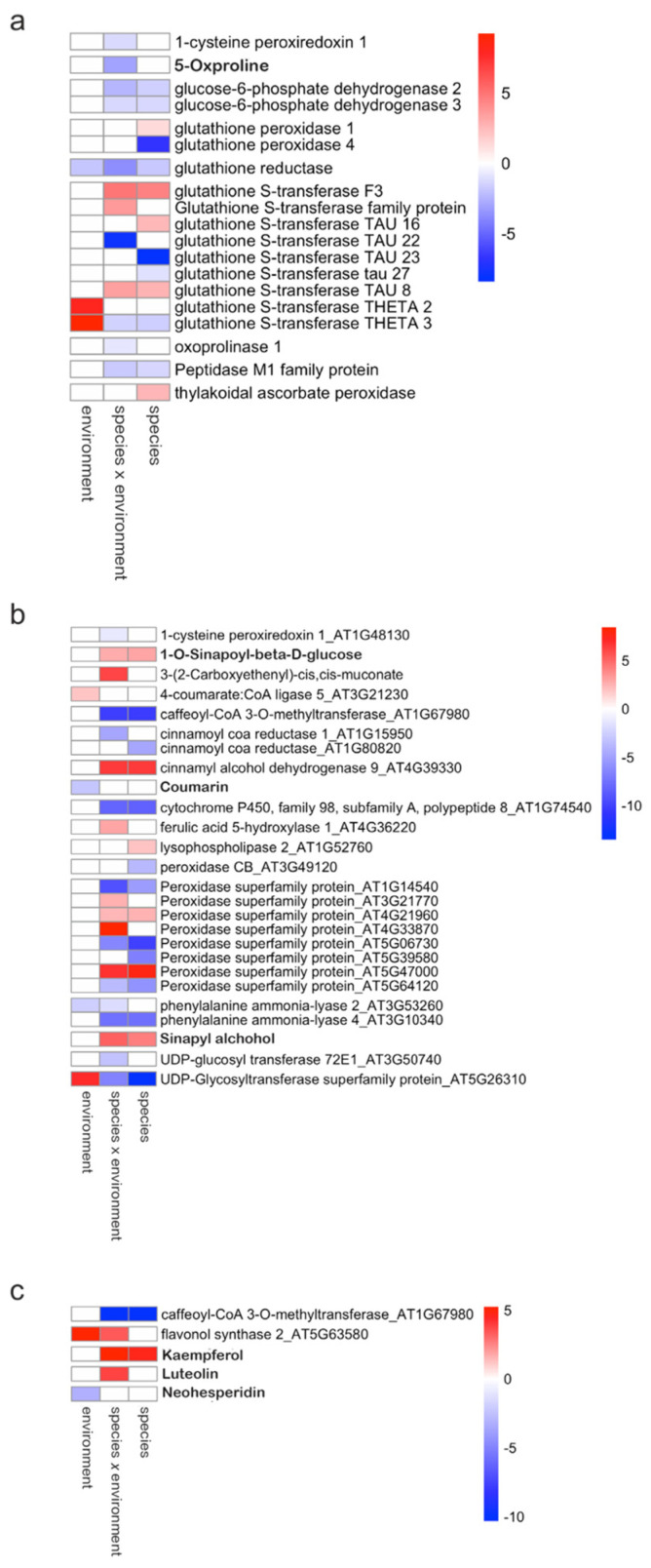
Heatmap of fold changes for differentially expressed genes and differentially accumulated metabolites (in bold) in (**a**) the glutathione metabolism pathway, (**b**) the phenylpropanoid biosynthesis pathway, and (**c**) the flavonoid biosynthesis pathway in for between-species (Np vs. Nc), environment (Ze vs. Lo), and species × environment (Np_Ze vs. Nc_Loc) comparisons. Colors represent log2 fold change: red—up-regulated; blue—down-regulated.

**Figure 6:**
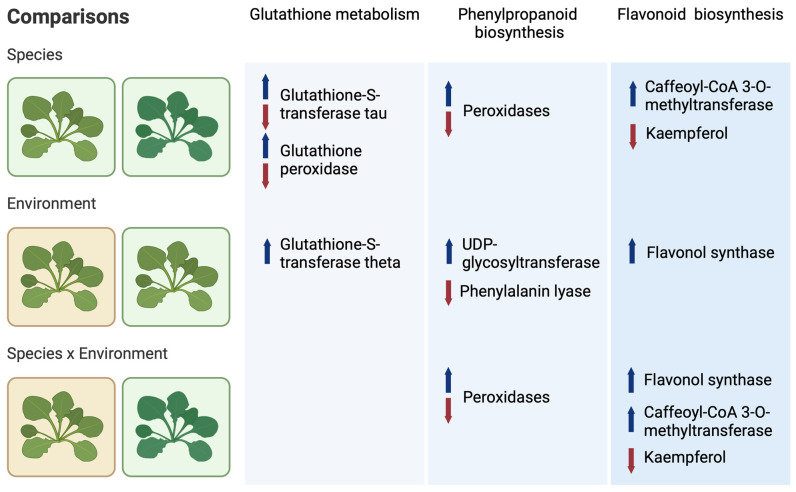
Main results of the integrated view on transcriptomics and metabolomics of studied *Noccaea* species for between-species (Np vs. Nc), environment (Ze vs. Lo), and species × environment (Np_Ze vs. Nc_Loc) comparisons.

The genes that showed differences between *Noccaea* populations included several glutathione-S-transferases (GSTs; Figure 5a, Figure 6 and Figures S6–S8). The majority belonged to the tau group of five GST genes, followed by two genes in the theta GSTs and two unclassified GSTs. The species comparison revealed the down-regulation of two tau GSTs and the up-regulation of two others. The environment comparison showed the up-regulation of theta group GSTs (Figure 5a and Figure 6) [18,19].

In addition to GST, we identified one glutathione reductase (GR), two glutathione peroxidases (GPx), two glucose-6-phosphate dehydrogenases, one oxoprolinase, a peptidase M1 family protein, 1-cysteine peroxiredoxin, thylakoidal ascorbate peroxidase, and 5-Oxoproline as DEGs and DAMs within the glutathione metabolism pathway. Glutathione reductase (GR) was down-regulated in *N. praecox* at the polluted site compared to *N. praecox* and *N. caerulescens* at the non-polluted site.

### 2.6. Phenylpropanoid Biosynthesis

The DEGs with significant differences between *N. praecox* and *N. caerulescens* at the non-polluted site were as follows: caffeoyl-CoA 3-O-methyltransferase (down-regulated), cinnamoyl-CoA reductase (down-regulated), cinnamyl-alcohol dehydrogenase (up-regulated), cytochrome P450 family 98 subfamily A8 (down-regulated), peroxidases (three up-regulated and three down-regulated), phenylalanine ammonia-lyase (down-regulated), and UDPglucose:coniferyl-alcohol 4′-beta-D-glucosyltransferase (down-regulated) (Figure 5b, Figure 6 and Figures S9–S11). Two metabolites were up-regulated: 1-O-sinapoyl-beta-D-glucose and sinapyl alcohol. The comparison between *N. caerulescens* and *N. praecox* from the polluted site yielded a similar pattern with some notable differences: up-regulation of 3-(2-Carboxyethenyl)-cis,cis-muconate and a peroxidase. The genes and metabolites with significant differences between *N. praecox* from the polluted site and non-polluted site in phenylpropanoid biosynthesis were a phenylalanine lyase, 4-coumarate: CoA ligase, UDP-glucose:coniferyl-alcohol 4′-beta-D-glucosyltransferase, and coumarin.

### 2.7. Flavonoid Biosynthesis

In the flavonoid biosynthesis pathway, we observed two DEGs, caffeoyl-CoA 3-O-methyltransferase and flavonol synthase, alongside three DAMs, kaempferol, luteolin, and neohesperidin (Figure 5c, Figure 6 and Figures S12–S14). Comparison between *N. praecox* and *N. caerulescens* showed an under-regulation of caffeoyl-CoA 3-O-methyltransferase, which seems typical for this species regardless of their metal exposure status. In contrast, flavonol synthase was up-regulated in *N. praecox* growing at the polluted site compared to *N. caerulescens* or *N. praecox* from the non-polluted site. In addition, kaempferol displayed an increased accumulation in *N. praecox* compared to *N. caerulescens*, regardless of the metal exposure.

## 3. Discussion

### 3.1. Transcriptome and Metabolome Analysis

Comparison of DEGs between individual locations and plant species showed that polluted conditions induced the expression of several genes (117) that were unique to the *N. praecox* population from the polluted site, with an additional 266 that were common with *N. caerulescens*, thus indicating that *N. praecox* activated the expression levels of these genes to cope with the metal stress. This is supported by the pathways analysis that shows the enrichment of pathways are linked to the detoxification of reactive oxygen species under polluted conditions, which is likely connected to the ability of *N. praecox* to withstand the impact of metal stress [20,21].

Analysis of DAMs showed that similar DAMs were up-regulated and down-regulated when *N. praecox* from the polluted site was compared to *N. caerulescens* that grows on non-polluted sites or if both *N. praecox* populations were compared. Among the DAMs that showed accumulation under polluted conditions were organic acids and flavonoids. Organic acids play an important role in the uptake and chelation of metals in plants. In leaves, metal binding in cell cytosol by organic ligands, such as organic acids, and their sequestration and immobilization in the vacuoles is considered one of the most important mechanisms of metal tolerance in plants [22]. In the leaves of *N. caerulescens* [23] and *Arabidopsis halleri* [24], Zn is sequestered predominantly in the vacuoles of epidermal or mesophyll cells, where it is coordinated mainly with malate. This is in line with our results, which showed an accumulation of malate in *N. praecox* leaves under polluted conditions. Additionally, flavonoids, comprising flavones, flavanols, flavanones, isoflavones, and anthocyanins, represent a widespread group of polyphenol compounds recognized for their non-enzymatic antioxidant properties [25]. Research has demonstrated the crucial role of flavonoids in enhancing plant tolerance to environmental stresses like heavy metals and drought [4]. Consequently, DAMs identified in the comparisons can serve as potential markers for understanding the response of *N. praecox* to heavy metal stress.

### 3.2. Integrated View on Transcriptomics and Metabolomics

An integrated view of transcriptomic and metabolomic analysis identified several pathways that showed differences in expression levels between *N. praecox* and *N. caerulescens* when growing at the same site. As the plants were growing in the same environmental conditions and in close proximity, this emphasizes the importance of studies on different hyperaccumulator species, as differences can be observed even between phylogenetically very closely related species such as *N. praecox* and *N. caerulescens* [26]. In addition, several pathways showed up-regulation in *N. praecox* under polluted conditions. Among these up-regulated pathways, several are commonly associated with metal stress, such as glutathione metabolism, phenylpropanoid biosynthesis, and flavonoid biosynthesis [4,27,28].

In plants, glutathione (GSH) is involved in detoxification and signaling pathways related to defense against biotic and abiotic stresses [27]. The synthesis of specific chelators and subsequent sequestration of metal complexes are of major importance under metal stress, with GSH serving as a key component due to its high metal-binding affinity and its role as a precursor of phytochelatins [29]. Therefore, it is expected to form a backbone for the detoxification of metal stress in plants. A detailed examination of glutathione metabolism in our study revealed differential expression patterns of GSTs in *N. praecox* compared to *N. caerulescens* at the non-polluted site, indicating potential species–specific preferences toward different isoenzymes. Interestingly, the tau group GSTs pattern did not differ much between *N. praecox* from different sites compared to *N. caerulescens*. In contrast, *N. praecox* showed a pronounced up-regulation of theta group GSTs when plants from polluted and non-polluted locations were compared. Although theta GSTs belong to the GSTs, they possess glutathione peroxidase (GPx) activity and are involved in reducing organic hydroperoxides produced during oxidative stress [18,19]. Other instances of GPx without assigned class showed differences in expression only if *N. caerulescens* was compared to *N. praecox* from the non-polluted site. It seems that an increase in demand for ROS removal in *N. praecox* under polluted conditions may prompt the activation of theta GST to cope with ROS formation and protect cells from oxidative damage. Importantly, in hyperaccumulating *P. americana*, the genes related to GST in the leaves were up-regulated when treated with Cd [13], meaning that the presence of metals triggers the expression of GSTs. Since Žerjav is a highly polluted area with former mining and smelting activities, it seems that the activated GST in *N. praecox* catalyzes the covalent binding of GHS with cytotoxic substrates to form glutathione S-conjugates, which are then sequestered in the vacuoles [30]. Although GSTs exist ubiquitously in plants, activation of specific GSTs is either species- or environment-specific.

In addition to GST, we identified a glutathione reductase (GR) that was down-regulated in *N. praecox* at the polluted site compared to *N. praecox* and *N. caerulescens* at the non-polluted site. This down-regulation can result in up to 60% reduction of the GSSG/GSH ratio under polluted conditions [31,32]. The decline in the GSSG/GSH ratio is due to the massive utilization of GSH for metal chelation. In *Arabidopsis thaliana* treated with Cd [31], it was observed that this decrease was accompanied by increased GR activity and, similarly in our study, a decrease in GR transcript levels. Thus, the mechanism of the metal homeostasis maintenance in hyperaccumulating *N. praecox* appears to be similar to *A. thaliana* in this respect.

As such, our results suggest similar glutathione metabolism as other plants and closely related hyperaccumulating species, with the significant difference of theta GSTs, which are used in *N. praecox* to cope with the increased ROS production under metal-enriched conditions, which is, to the best of our knowledge, a unique and novel trait of this hyperaccumulator. Nevertheless, to establish the exact function in regard to ROS management, a controlled experiment should be performed to rule out other possible causes of ROS formation under natural conditions.

Several DEGs and DAMs were also observed in the phenylpropanoid pathway when comparing *N. praecox* from polluted and non-polluted sites. The activation of the phenylpropanoid biosynthetic pathway under abiotic stress conditions leads to the accumulation of various phenolic compounds capable of scavenging harmful reactive oxygen species [28]. This finding is consistent with the previous study demonstrating the up-regulation of phenylpropanoid biosynthesis genes in response to Cd stress in cotton [33]. Nevertheless, our results suggest that changes in gene expression and metabolite accumulation in phenylpropanoid biosynthetic pathways of *N. praecox* were species specific and showed only minimal perturbation due to metal enrichment in the environment.

In addition, analysis of the flavonoid biosynthesis pathway suggests that *N. praecox* exhibits increased kaempferol accumulation, which serves as both an antioxidant and a precursor for synthesizing other flavonoids. The trait seems to be species specific and activated regardless of metal pollution. Apart from the already mentioned roles, kaempferol is also involved in hormone regulation [34], meaning that its role is also important at non-polluted sites. Flavonoids are an important group of non-enzymatic antioxidants [25] that are already proven to enhance plant tolerance to metal stress [4]. For instance, Ref. [35] showed that polyphenolic compounds in *Sedum caespitosum*, including quercetin, kaempferol, and myrcetin, conferred protection against H_2_O_2_-induced cytotoxicity. Moreover, studies indicate that exogenous quercetin [36] and rutin (a quercetin glycoside [37]) alleviated symptoms of metal stress in Cd-treated plants.

The heightened accumulation of flavonoids signifies a typical plant response to stress, with flavonoid synthesis activated under oxidative injury. The observed up-regulation of flavonol synthase (FLS) in *N. praecox* from the polluted site may provide the plant with an additional reservoir of flavonoids that exert a protective effect against reactive oxygen species (ROS) generated by metal uptake. It is likely that the synthesis of flavonols arises in the presence of metals, as it was demonstrated that the content of flavonols increases under heavy metal treatment and also depends on the metal [38]. It seems to be the trait that is triggered by metal concentration, and it is not species specific. In transgenic *A. thaliana*, for example, maize FLS (ZmFLS1), overexpression resulted in a higher ROS scavenging rate and lower oxidative damage [39]. Similar up-regulation of FLS was also observed in *Salix matsudana* after Cd exposure [40], suggesting a common response to metal stress. As such, the induced accumulation of flavonoids seems to be an additional key difference in the tolerance strategy of *N. praecox* compared to the well-known and closely related species *N. caerulescens*.

## 4. Materials and Methods

### 4.1. Site Description and Sample Collection

During the flowering phase, three naturally occurring *N. caerulescens* (Nc) and *N. praecox* (Np) plants were collected at Lokovec (Lo; 46°2′39.2706″ N, 13°46′8.9934″ E), and three naturally occurring *N. praecox* plants were collected in Žerjav (Ze; 46°28′26.1258″ N, 14°51′56.0118″ E). Plants were transferred to the lab. Lokovec is a non-polluted grassland, whereas Žerjav is a polluted grassland site due to centuries-long mining and smelting activity [26]. The soil at the polluted site contains, on average, 38 mg Cd kg^−1^, 220 mg Zn kg^−1^, and 9078 mg Pb kg^−1^, whereas the concentrations at the non-polluted site Lokovec were much lower (1.1 mg Cd kg^−1^, 10 mg Zn kg^−1^, and 117 mg Pb kg^−1^) [26]. The soils at both sites belong to the reference group of phaeozems with calcareous rocks, namely limestone in Lokovec and dolomite in Žerjav, as the parent material. Both soils are eutric, with a pH of 6.8–6.9.

### 4.2. Inductively Coupled Plasma Mass Spectrometry (ICP-MS)

The concentrations of Zn, Cd, and Pb in dried leaves were determined following acid digestion by inductively coupled plasma mass spectrometry (ICP-MS). Dried samples were milled to a fine powder using a mortar and a pestle, and accurately weighed powdered material was digested in closed vessels using a microwave digester (MARS Xpress, CEM Microwave Technology, Cologno Al Serio, Italy). Samples were digested with 6 mL of concentrated HNO_3_ and 2 mL of 30% H_2_O_2_ at 180 °C (holding for 60 min). Digested samples were diluted to 30 mL with MilliQ water before element analyses. Total Zn, Cd, and Pb concentrations of digested samples were determined by ICP-MS (Nexion 1000, PerkinElmer, Waltham, MA, USA). Blank digestions were performed to determine background concentrations of elements, and a tomato leaf standard (Reference 1573a; National Institute of Standards and Technology, NIST, Gaithersburg, MD, USA) was used as an analytical control.

### 4.3. Transcriptome Analysis

Three plants from each population were selected for transcriptome analysis. A healthy rosette leaf was collected from each plant, flash-frozen in liquid nitrogen, and stored at −80 °C until RNA extraction. For the transcriptome analysis, we followed extraction protocol and sequencing conditions as described previously [41]: sequencing was performed by the Macrogen company using the TruSeq Stranded mRNA Sample Preparation Kit (Illumina, San Diego, CA, USA) and the Illumina NovaSeq6000 platform (paired-end sequencing with 150 nt reads). Similarly, the transcriptome assembly and annotation followed the same procedure as in [41]. In short, raw reads were processed with RCorrector v1.0.521 [42] and TrimGalore v0.6.2 (https://github.com/FelixKrueger/TrimGalore, accessed on 15 May 2024) to remove uncorrectable reads and adaptors and quality trimming. Ribosomal RNAs potentially still present after polyA capture were removed through alignment against the SILVA Ribosomal database (Release 138) with Bowtie2 v2.5.122 [43]. Transcriptome was assembled de novo using Trinity v2.13.224 [44] with a minimum length of contigs set to 300 nt. The quality of the assembly was analyzed with TrinityStats.pl, and the transcriptome completeness was estimated using the Benchmarking Universal Single-Copy Orthologs (BUSCO) v525 [45] against the conserved single-copy Viridiplantae genes database on the server gVolante [https://gvolante.riken.jp] (accessed on 25 October 2024). Finally, filtered reads were mapped back to the transcriptome to evaluate individual mapping rate with Bowtie2, and ExN50 was generated by Trinity accessory scripts.

The assembly retained the sample information, and the Kallisto pseudoaligner [46] was used to map the original sequence reads to the assembly to quantify the transcripts.

Transcriptome assembly annotation was performed using the Trinotate v.4.0.0 pipeline [http://trinotate.github.io] (accessed on 21 May 2024). The original RNA sequencing data have been deposited at the NCBI Sequence Read Archive under the SRA study accession SRR28777001-SRR28777009.

### 4.4. Metabolome Analysis

Three plants from each population were used for metabolome analysis. A healthy rosette leaf was collected from each plant, flash-frozen in liquid nitrogen for at least 15 min, and crushed into smaller pieces. The samples were then freeze-dried for three days at −96 °C and 0.0012 mbar (Coolsafe (Scanvac) LaboGene, Allerød, Denmark) and stored at −80 °C before being sent to the BGI company for untargeted metabolomic analysis.

For the analysis, a 50 mg sample from each plant was extracted with 800 μL of pre-cooled extraction solution (methanol: H_2_O = 7:3, *v*/*v*) and 20 μL Internal Standard (d3-Leucine, 13C9-Phenylalanine, d5-Tryptophan, and 13C3-Progesterone). Homogenization was conducted using a weaving grinder and ultra-sonification. After one hour of incubation at −20 °C, the extracts were centrifuged at 14,000 rpm (4 °C, 15 min).

The separation and identification of metabolites were performed using Waters UPLC I-Class Plus (Waters, Milford, MA, USA) tandem Q Exactive high-resolution mass spectrometer (Thermo Fisher Scientific, Waltham, MA, USA). Chromatographic separation was performed using Hypersil GOLD aQ Dim column (1.9 μm, 2.1 × 100 mm; Thermo Fisher Scientific, Waltham, MA, USA) at 0.3 mL/min and 40 °C. The mobile phase consisted of solvent A (0.1% formic acid in water) and solvent B (0.1% formic acid in acetonitrile). The initial mobile phase composition was maintained at 5% solvent B for 2 min, changed to 95% B (2–22 min) and held for 5 min (22–27 min). The injection volume was five μL. The scan range was 125~1500 m/z for positive ions and 100–1500 *m*/*z* for negative ions, with a resolution of 70,000 for MS acquisitions. For MSMS acquisition, 30,000 was used. The fragmentation energy was 20, 40, and 60 eV. The sheath gas flow rate and aux gas flow rate were 40 and 10, respectively. The spray voltage of the positive ion mode and negative ion mode was 3.80 kV and 3.20 kV, respectively. Ion capillary temperature and aux gas heater temperature were 320 °C and 350 °C, respectively.

Data preprocessing was performed using metaX [47]. Classification and functional annotation analysis via KEGG and HMDB databases were performed to obtain KEGG ID, HMDB ID, category, and KEGG Pathway.

### 4.5. Statistics

All analyses were performed in R v4.3.0. Kruskal–Wallis with Dunn’s test used to compare the leaf-accumulated metals between different *Noccaea* populations. TrinotateR (https://github.com/cstubben/trinotateR, accessed on 9 June 2024) was used to import the transcriptome assembly. DESeq2 (v1.40.2, [48]) was used for differential gene expression analysis (*p* < 0.05). Genes with the absolute value of |log2 fold change| > 1 were deemed differentially expressed (DEG). PCA and OPLS-DA were utilized to identify significantly different metabolite levels (*p* < 0.05) using the metaX (v1.0.3, [47]) library. Metabolites were designated as differentially altered metabolites (DAMs) if log2 fold change ≥1 or ≤0.65, along with variable importance in projection (VIP) scores > 1. Library clusterprofiler (v4.8.3, [49]) was used for enrichment analysis. Libraries ggplot2 (v3.4.4, [50]), ggvenn (v0.1.10, 10.32614/CRAN.package.ggvenn, accessed on 9 June 2024), pheatmap (v1.0.12, https://github.com/raivokolde/pheatmap, accessed on 9 June 2024), and pathview (v1.40.0, [51]) were used for visualization of the results.

## 5. Conclusions

The comparison between *N. praecox* populations from polluted and non-polluted sites, along with the co-occurring *N. caerulescens* at the non-polluted site, enabled us to assign transcriptomic and metabolomic changes to the metal status or species per se. A few species–specific traits connected to the detoxification of metals and metal-induced oxidative stress that advance our current knowledge of hyperaccumulation mechanisms in *Noccaea* species were discovered. As these differences were observed despite the relative evolutionary closeness of both species [26], our result emphasizes the importance of further expanding our knowledge on hyperaccumulators to potentially exploit their mechanisms for phytoremediation efforts or food quality improvements.

## Figures and Tables

**Table 1:** Leaf concentrations of Cd, Zn, and Pb in *N. praecox* and *N. caerulescens* plants at the polluted site (Žerjav) and the non-polluted site (Lokovec). Different letters depict statistically significant difference at *p* < 0.05. DWdry weight.

Population	Cd (mg kg^−1^ DW)	Zn (mg kg^−1^ DW)	Pb (mg kg^−1^ DW)
*N. praecox* Žerjav	1223 ± 10 a	4271 ± 23 a	107 ± 4.4 a
*N. praecox* Lokovec	125 ± 4.8 b	1004 ± 2.8 b	0.65 ± 0.17 b
*N. caerulescens* Lokovec	252 ± 2.4 b	4422 ± 12 a	0.93 ± 0.02 b

## Data Availability

Metabolome data tables were deposited at Zenodo (10.5281/zenodo.10991915).

## Supplementary Materials

The following supporting information can be downloaded at: https://www.mdpi.com/article/10.3390/plants13223149/s1, Figure S1: Principal component analysis (PCA) ordination of metabolomes from leaves of *Noccaea praecox* (Np) and *N. caerulescens* (Nc) from the polluted (Ze) and non-polluted site (Lo); Figure S2: KEGG enrichment analysis for metabolites for comparisons of (a) species, (b) environment, and (c) species × environment; Figure S3: KEGG pathway enrichment analysis of DEGs and DAMs in transcriptomics and metabolomics comparing *N. praecox* from polluted and non-polluted sites (environment). The y-axis represents KEGG metabolic pathways, and the x-axis represents the enriched −log(*p*-value) for DEGs and DAMs; Figure S4: KEGG pathway enrichment analysis of DEGs and DAMs in transcriptomics and metabolomics comparing *N. praecox* from polluted and *N. caerulescens* from non-polluted sites (environment). The y-axis represents KEGG metabolic pathways, and the x-axis represents the enriched −log(*p*-value) for DEGs and DAMs; Figure S5: KEGG pathway enrichment analysis of DEGs and DAMs in transcriptomics and metabolomics comparing *N. praecox* and *N. caerulescens* from polluted and non-polluted sites (environment). The y-axis represents KEGG metabolic pathways, and the x-axis represents the enriched −log(*p*-value) for DEGs and DAMs; Figure S6: KEGG glutathione metabolism pathway for comparison between *Noccaea praecox* and *N. caerulescens* at the non-polluted site (species). The colors represent the enrichment of transcripts; Figure S7: KEGG glutathione metabolism pathway for comparison between *Noccaea praecox* from the polluted site and *N. caerulescens* from the non-polluted site (species × environment). The colors represent the enrichment of transcripts (blue–gold) and metabolites (red–green); Figure S8: KEGG glutathione metabolism pathway for comparison between *Noccaea praecox* from the polluted site and from the non-polluted site (species × environment). The colors represent the enrichment of transcripts (blue–gold); Figure S9: KEGG phenylpropanoid biosynthesis pathway for comparison between *Noccaea praecox* and *N. caerulescens* at the non-polluted site (species). The colors represent the enrichment of transcripts (blue–gold) and metabolites (red–green); Figure S10: KEGG phenylpropanoid biosynthesis pathway for comparison between *Noccaea praecox* from the polluted site and *N. caerulescens* from the non-polluted site (species × environment). The colors represent the enrichment of transcripts (blue–gold) and metabolites (red–green); Figure S11: KEGG phenylpropanoid biosynthesis pathway for comparison between *Noccaea praecox* from a polluted site and from a non-polluted site (species × environment). The colors represent the enrichment of transcripts (blue–gold); Figure S12: KEGG flavonoid biosynthesis pathway for comparison between *Noccaea praecox* and *N. caerulescens* at the non-polluted site (species). The colors represent the enrichment of transcripts (blue–gold) and metabolites (red–green); Figure S13: KEGG flavonoid biosynthesis pathway for comparison between *Noccaea praecox* from the polluted site and *N. caerulescens* from the non-polluted site (species × environment). The colors represent the enrichment of transcripts (blue–gold) and metabolites (red–green); Figure S14: KEGG flavonoid biosynthesis pathway for comparison between *Noccaea praecox* from the polluted site and from the non-polluted site (species × environment). The colors represent the enrichment of transcripts (blue–gold) and metabolites (red–green).
