# Supplementary material for: Species-Specific and Pollution-Induced Changes in Gene Expression and Metabolome of Closely Related Noccaea Species Under Natural Conditions
Source: Plants (Basel). 2024 Nov 9;13(22):3149. doi: 10.3390/plants13223149 (PMC11597696; doi:10.3390/plants13223149)
Supplement: Supplementary file 1 [file plants-13-03149-s001.zip › plants-3285125-supplementary.pdf]

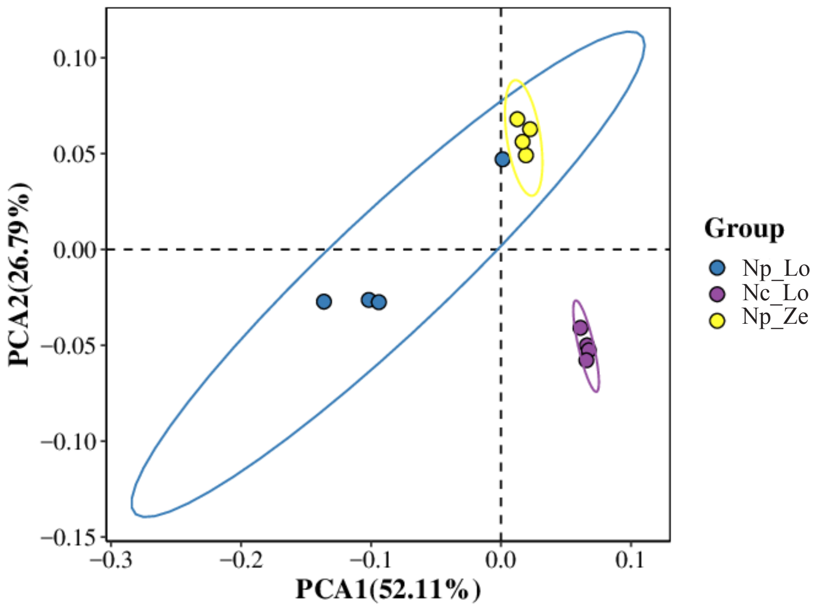

Supplementary Figure S1. Principal component analysis (PCA) ordination of metabolomes from leaves of *Noccaea praecox* (Np) and *N. caerulescens* (Nc) from the polluted (Ze) and non-polluted site (Lo).

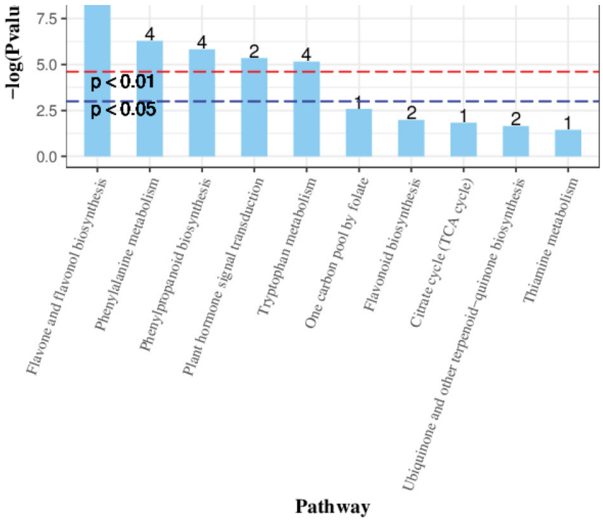

b

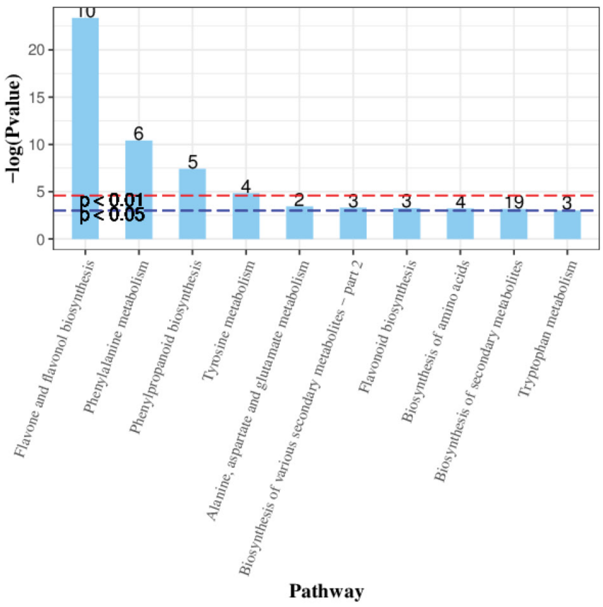

c

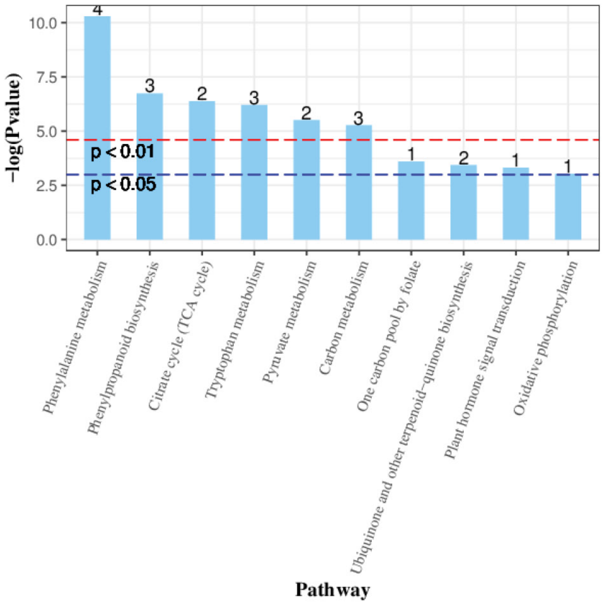

Supplementary Figure S2. KEGG enrichment analysis for metabolites for comparisons **a)** species, **b)** environment, and **c)** species *x* environment.

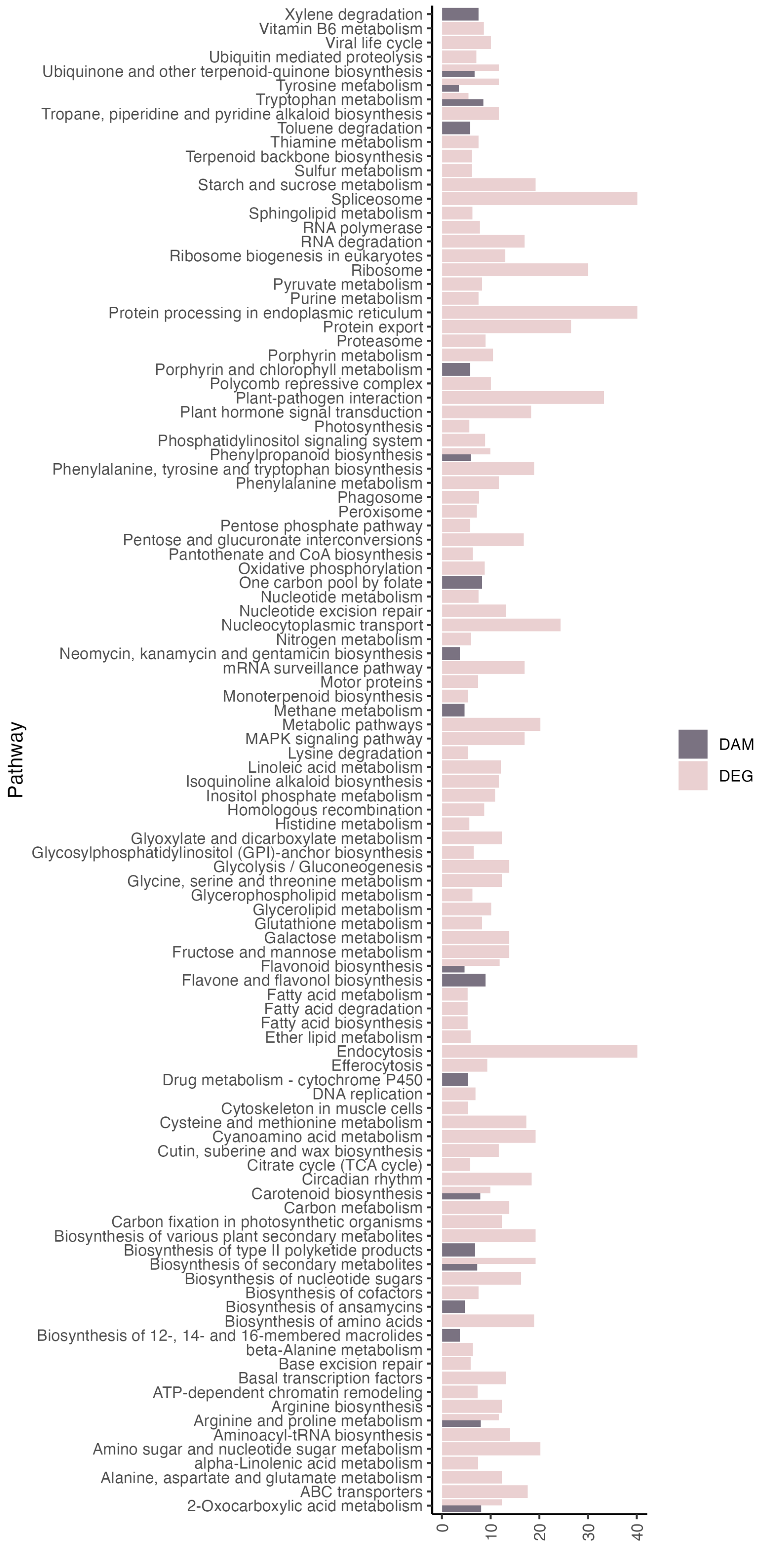

Supplementary Figure S3. KEGG pathway enrichment analysis of DEGs and DAMs in transcriptomics and metabolomics comparing *N. praecox* from polluted and non-polluted site (environment). The y-axis represents KEGG metabolic pathways, and the x-axis represents the enriched -log(p-value) for DEGs and DAMs.

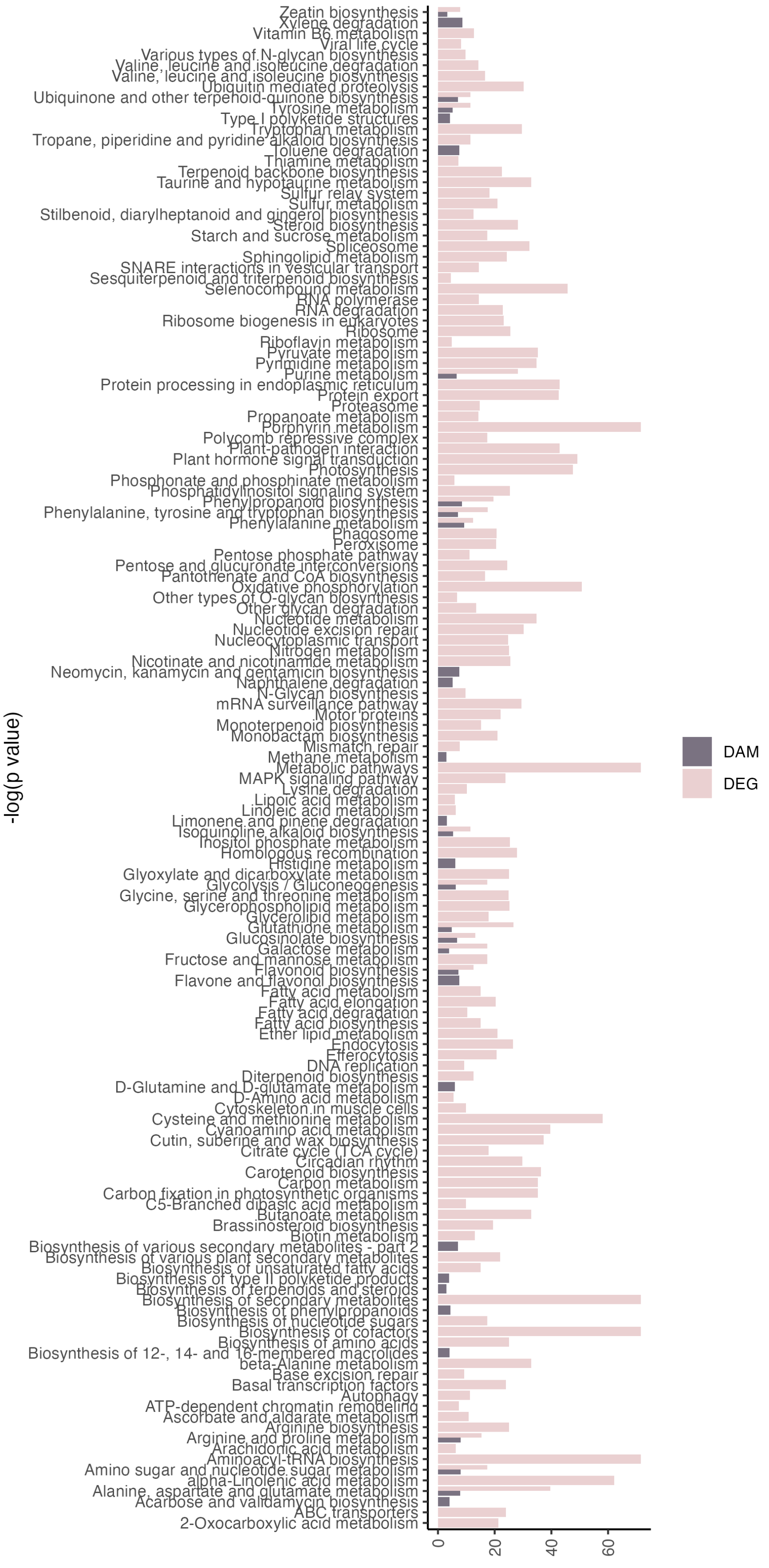

Supplementary Figure S4. KEGG pathway enrichment analysis of DEGs and DAMs in transcriptomics and metabolomics comparing *N. praecox* from polluted and *N. caerulescens* from non-polluted site (environment). The y-axis represents KEGG metabolic pathways, and the x-axis represents the enriched -log(p-value) for DEGs and DAMs.

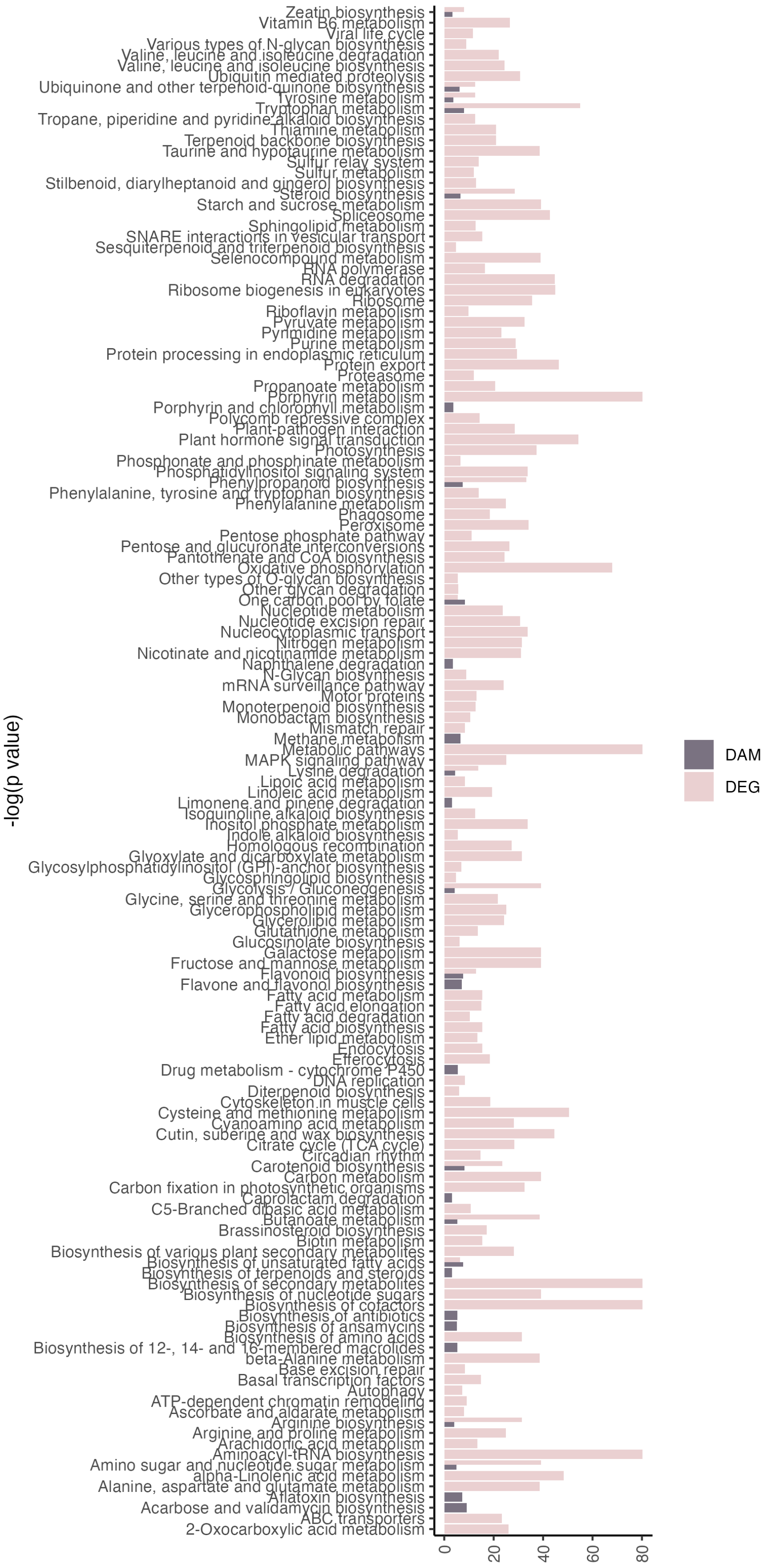

Supplementary Figure S5. KEGG pathway enrichment analysis of DEGs and DAMs in transcriptomics and metabolomics comparing *N. praecox* and *N. caerulescens* from polluted and non-polluted site (environment). The y-axis represents KEGG metabolic pathways, and the x-axis represents the enriched -log(p-value) for DEGs and DAMs.

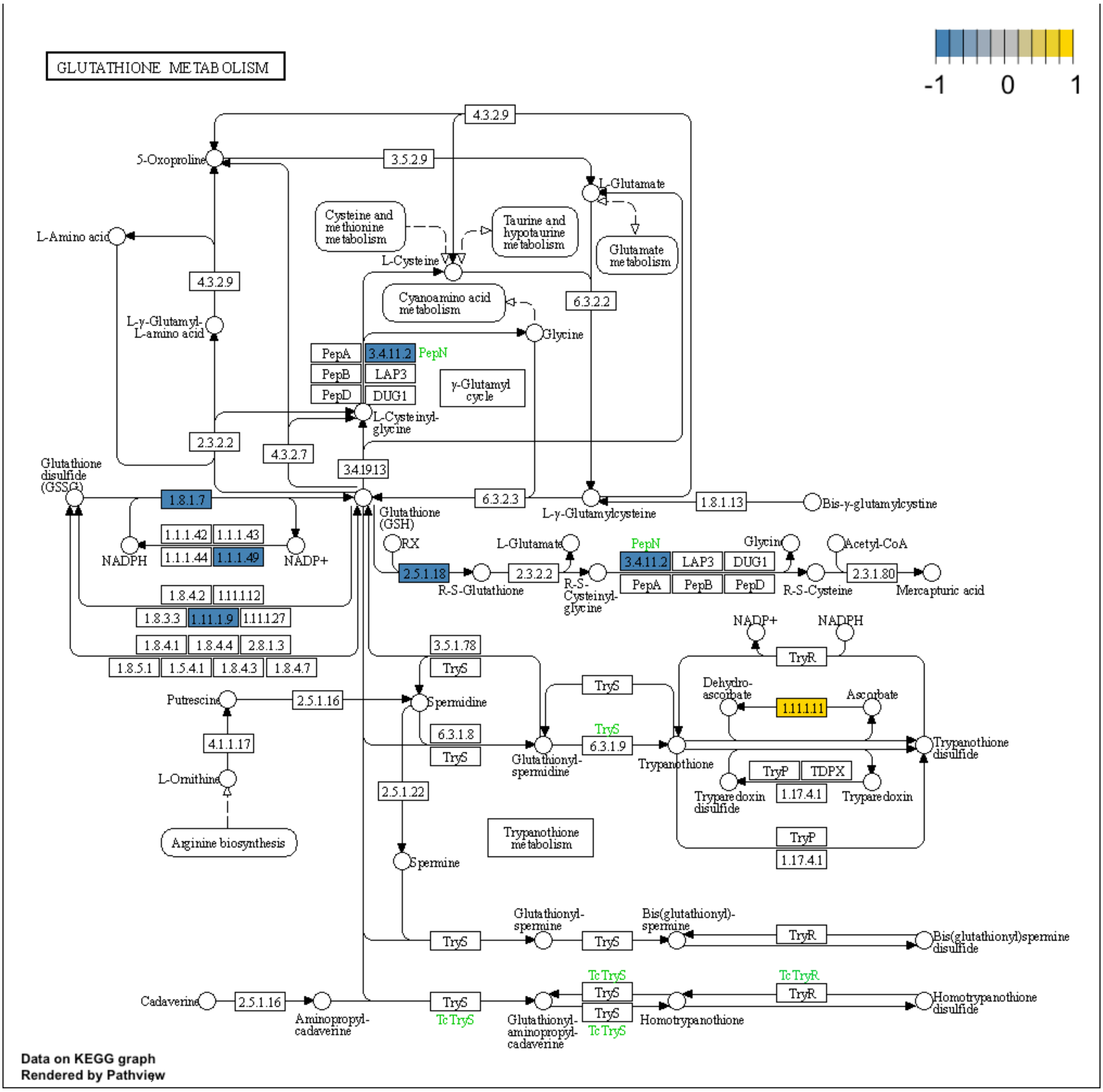

Supplementary Figure S6. KEGG glutathione metabolism pathway for comparison between *Noccaea praecox* and *N. caerulescens* at the non-polluted site (species). Colors represent enrichment of transcripts.

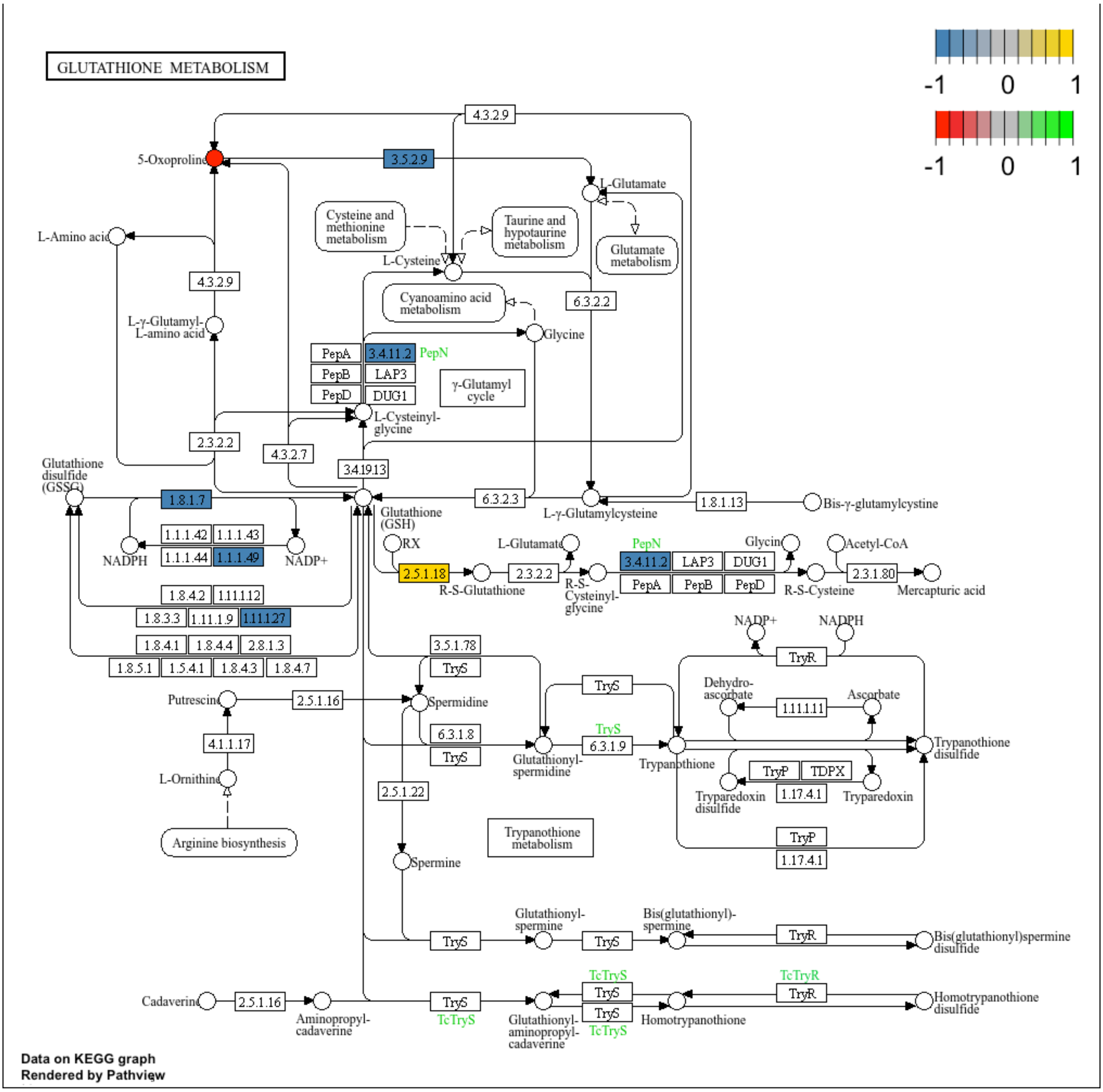

Supplementary Figure S7. KEGG glutathione metabolism pathway for comparison between *Noccaea praecox* from polluted site and *N. caerulescens* from the non-polluted site (species x environment). Colors represent enrichment of transcripts (blue-gold) and metabolites (red-green).

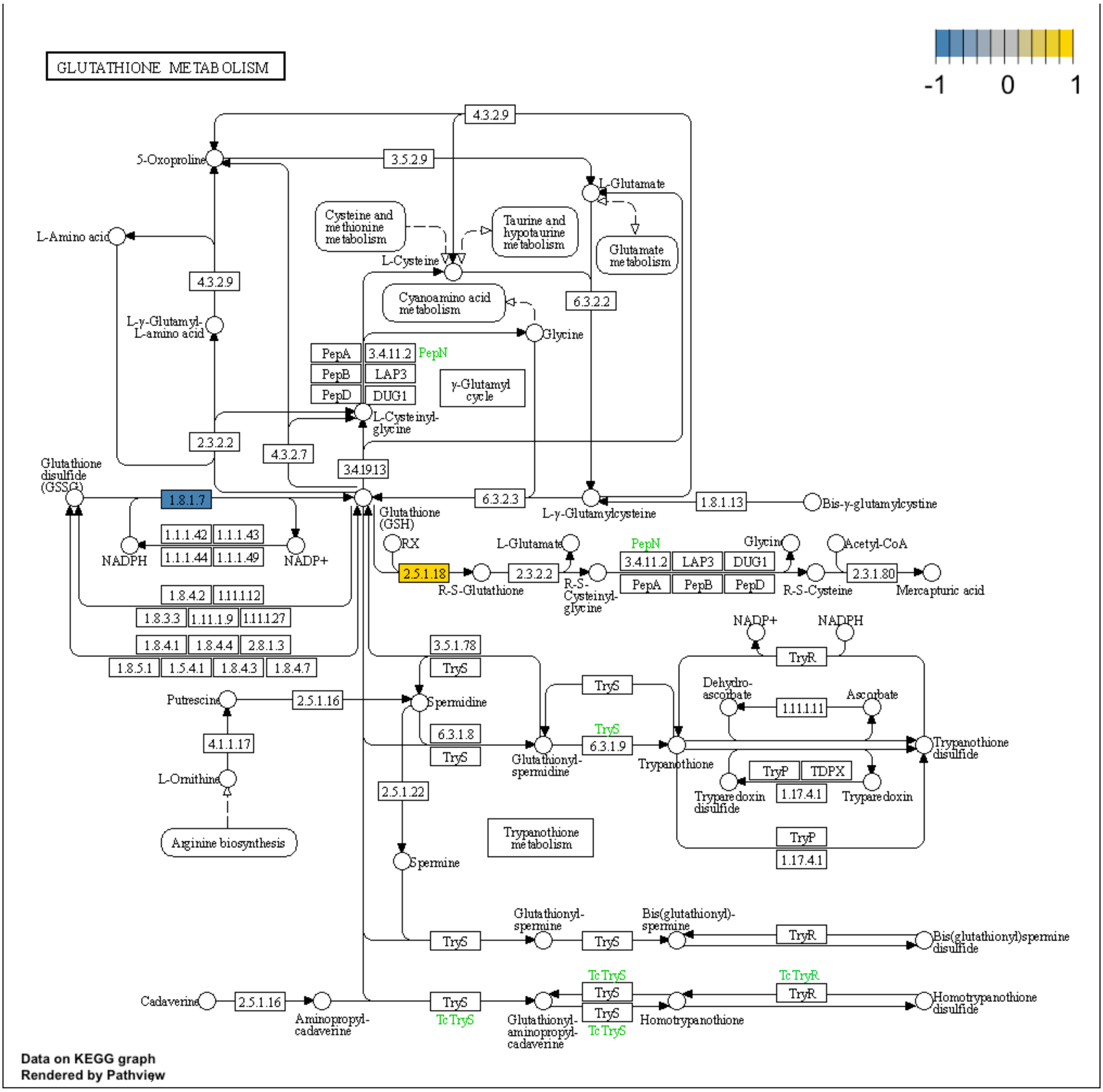

Supplementary Figure S8. KEGG glutathione metabolism pathway for comparison between *Noccaea praecox* from polluted site and from the non-polluted site (species x environment). Colors represent enrichment of transcripts (blue-gold).

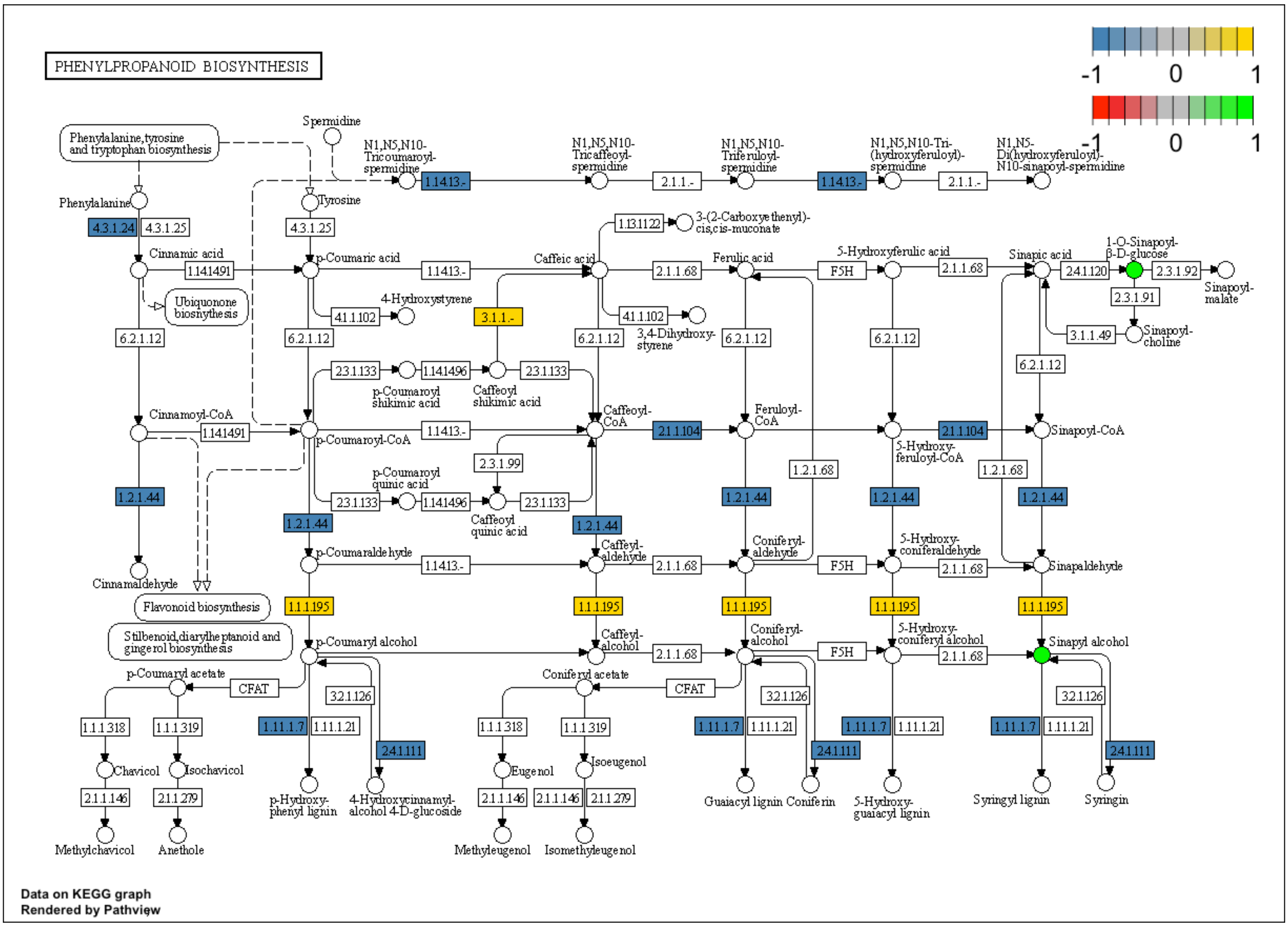

Supplementary Figure S9. KEGG phenypropanoid biosynthes pathway for comparison between *Noccaea praecox* and *N. caerulescens* at the non-polluted site (species). Colors represent enrichment of transcripts (blue-gold) and metabolites (red-green).

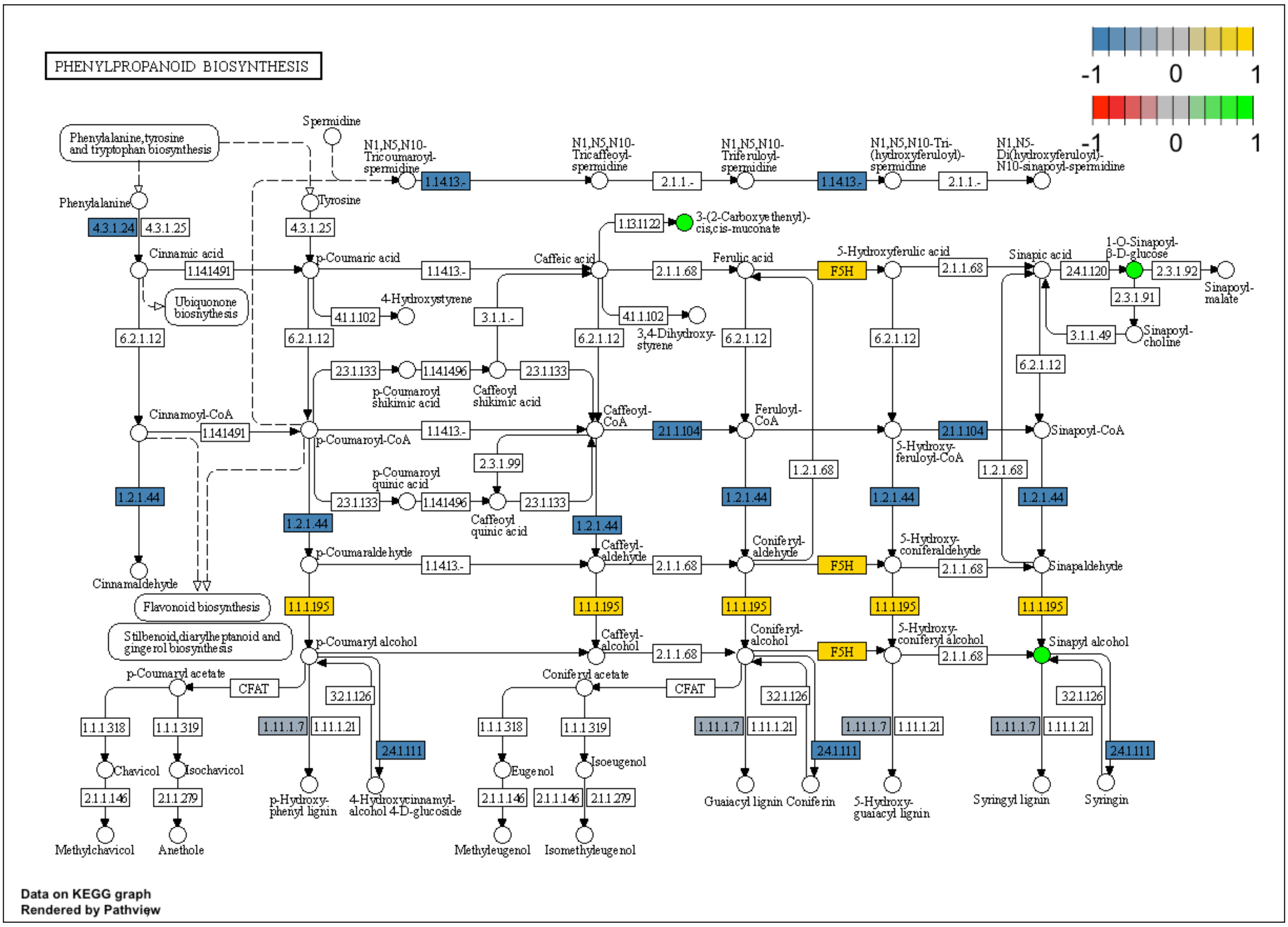

Supplementary Figure S10. KEGG phenylpropanoid biosynthesis pathway for comparison between *Noccaea praecox* from polluted site and *N. caerulescens* from the non-polluted site (species x environment). Colors represent enrichment of transcripts (blue-gold) and metabolites (red-green).

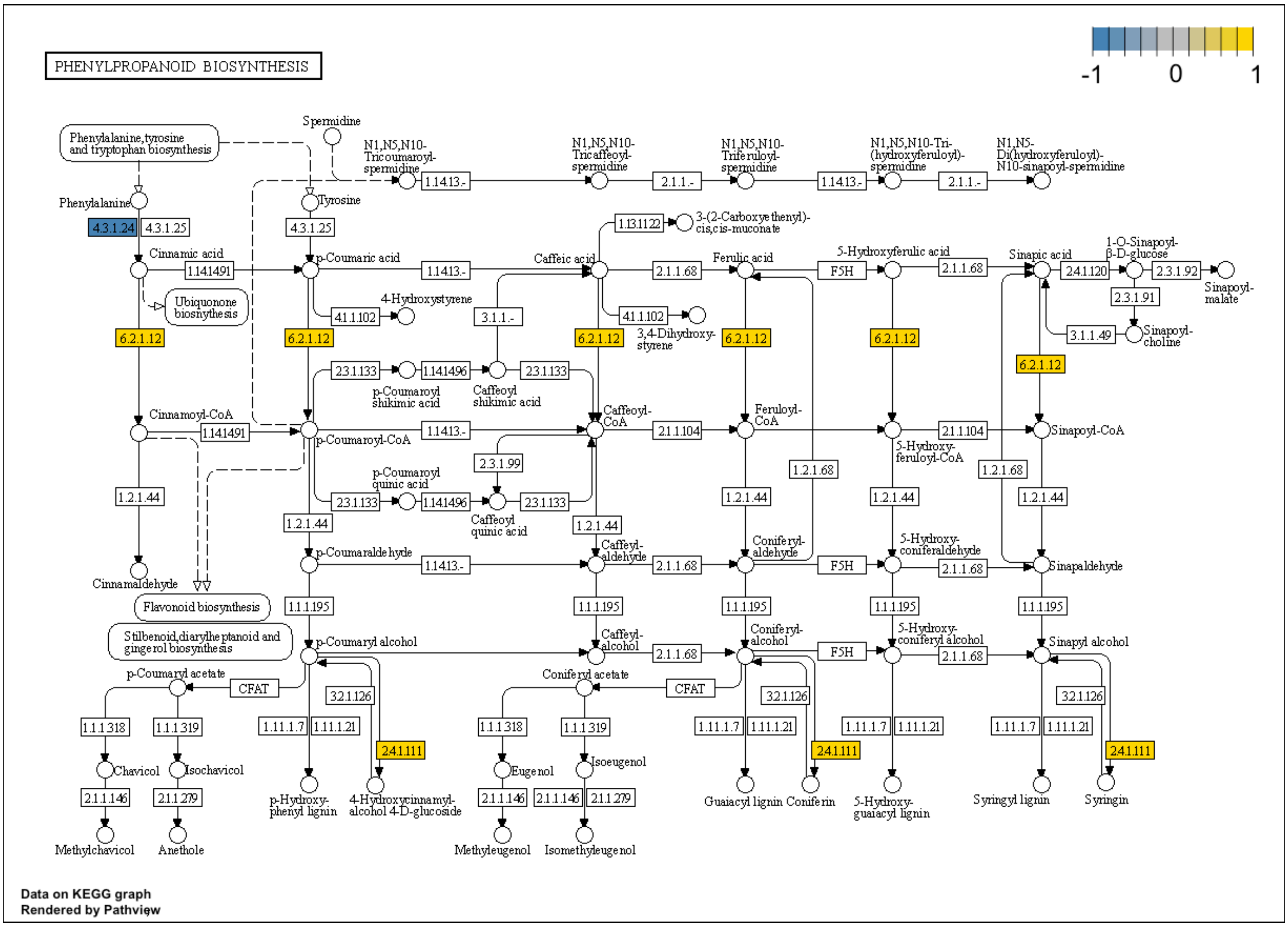

Supplementary Figure S11. KEGG phenylpropanoid biosynthesis pathway for comparison between *Noccaea praecox* from polluted site and from the non-polluted site (species x environment). Colors represent enrichment of transcripts (blue-gold).

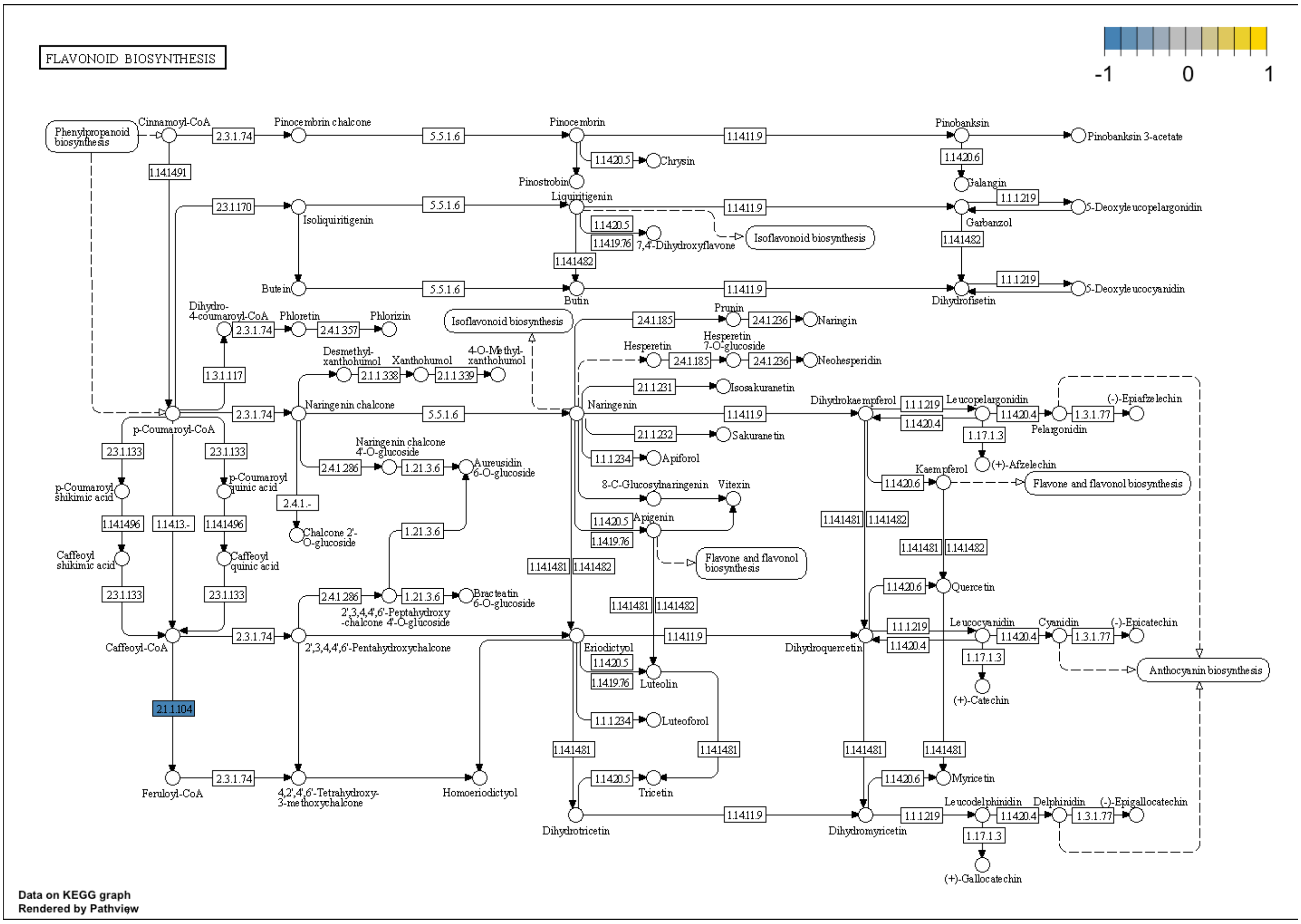

Supplementary Figure S12. KEGG flavonoid biosynthesis pathway for comparison between *Noccaea praecox* and *N. caerulescens* at the non-polluted site (species). Colors represent enrichment of transcripts (blue-gold) and metabolites (red-green).

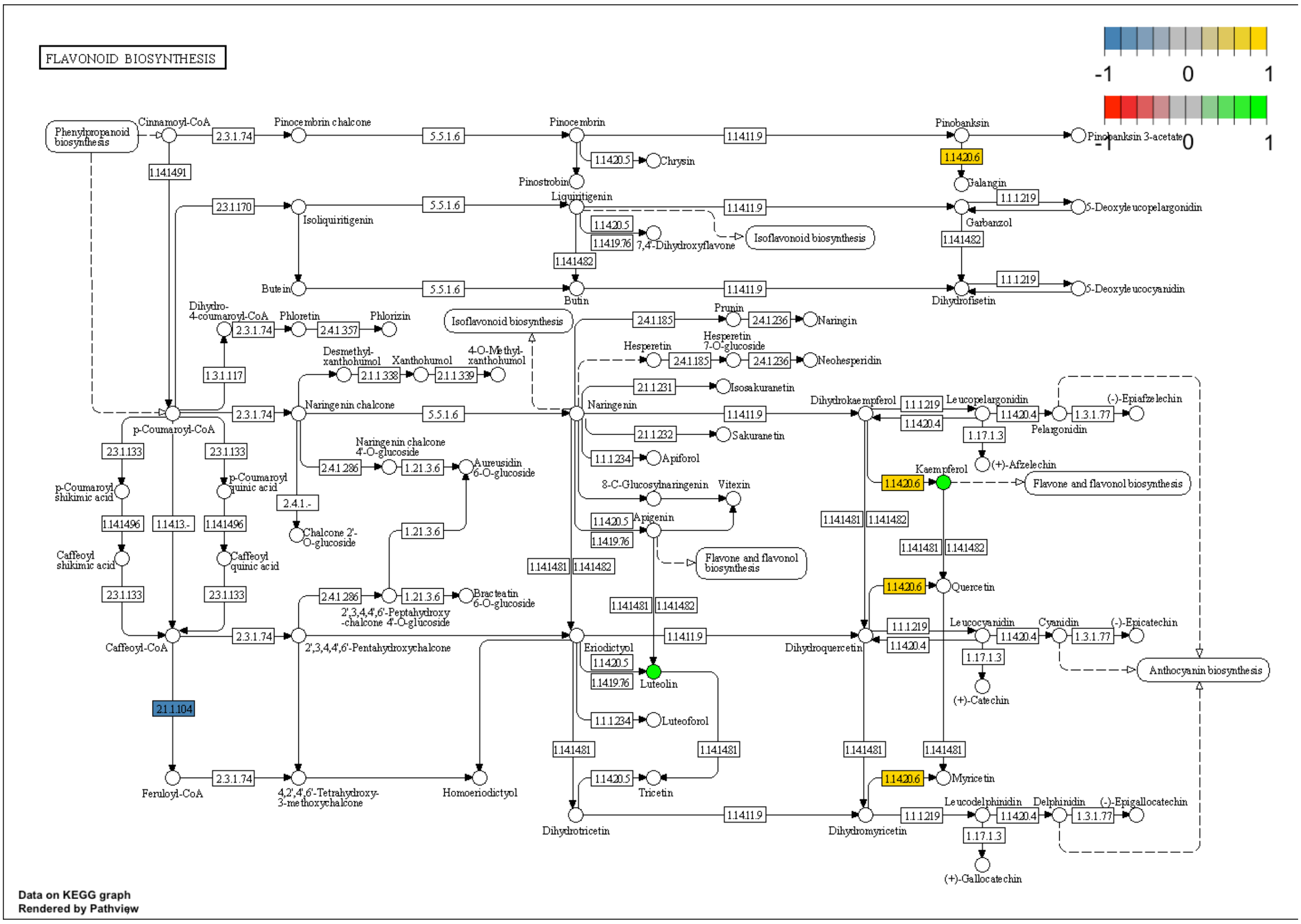

Supplementary Figure S13. KEGG flavonoid biosynthesis pathway for comparison between *Noccaea praecox* from polluted site and *N. caerulescens* from the non-polluted site (species x environment). Colors represent enrichment of transcripts (blue-gold) and metabolites (red-green).

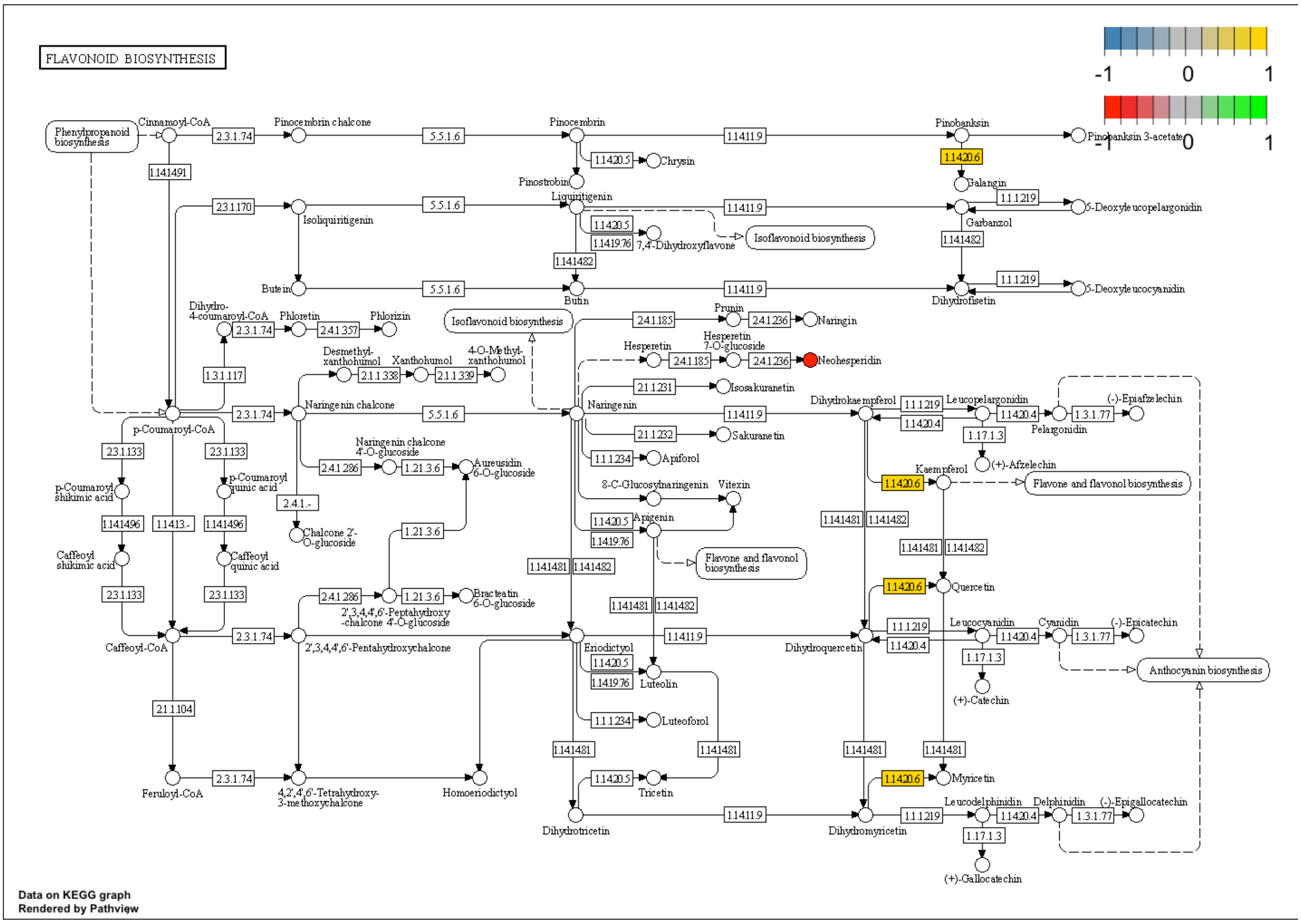

Supplementary Figure S14. KEGG flavonoid biosynthesis pathway for comparison between *Noccaea praecox* from polluted site and from the non-polluted site (species x environment). Colors represent enrichment of transcripts (blue-gold) and metabolistes (red-green).
